# Relationship between *β*1-AA and AT1-AA and Cardiac Function in Patients with Hypertension Complicated with Left Ventricular Diastolic Function Limitation

**DOI:** 10.1155/2023/7611819

**Published:** 2023-12-13

**Authors:** Liang Wang, Xue-Bai Lv, Yu-Ting Yuan, Ning Wang, Hong-Ying Yao, Wen-Chao Zhang, Peng-Fei Yin, Xiao-Hui Liu

**Affiliations:** ^1^Department of Cardiology, Peking University International Hospital, Beijing 102206, China; ^2^Third Medical Center, The General Hospital of the People's Liberation Army, Beijing 100039, China

## Abstract

**Objective:**

To investigate the association between *β*1 adrenergic receptor autoantibodies (*β*1-AA) and angiotensin II type-1 receptor autoantibodies (AT1-AA) and cardiac function in patients with hypertension complicated with left ventricular diastolic function limitation.

**Methods:**

A total of 120 patients with essential hypertension who were not taking drug treatment and were hospitalised in the Department of Cardiology at the authors' hospital from April 2018 to December 2018 were enrolled in this study and divided into a diastolic dysfunction group (65 cases) and a normal diastolic group (55 cases) according to their left ventricular diastolic function. The levels of cardiac parameters, *β*1-AA, AT1-AA, and other indicators were compared. Logistic regression analysis was used to analyse the related factors affecting left ventricular diastolic dysfunction (LVDD). The diagnostic efficacy of related factors in the diagnosis of diastolic dysfunction was evaluated.

**Results:**

Univariate analysis demonstrated that the left ventricular posterior wall diameter (10.29 ± 1.23 vs. 9.12 ± 1.53), left ventricular systolic dysfunction (10.56 ± 1.37 vs. 9.43 ± 1.44), systolic blood pressure (152.37 ± 10.24 vs. 140.33 ± 5.99), diastolic blood pressure (95.66 ± 6.34 vs. 87.33 ± 7.28), *β*1-AA (33 vs. 9 cases), and AT1-AA (35 cases vs. 12 cases) were higher in the dysfunction group than in the control group (all *P* < 0.05). Multivariate regression analysis showed that *β*1-AA (odds ratio (OR) = 1.96, 95% confidence interval (CI): 1.369–4.345) and AT1-AA (OR = 2.02, 95% CI: 1.332–6.720) were independent risk factors for cardiac diastolic dysfunction (*P* < 0.05). Both autoimmune antibodies had a certain predictive value, and the combined prediction value of the two was the highest, with an area under the curve of 0.942 (95% CI: 0.881~0.985).

**Conclusion:**

The positive rate of *β*1-AA and AT1-AA in essential hypertension patients with LVDD was higher than that in the normal group. Both *β*1-AA and AT1-AA could be used as early markers of LVDD in essential hypertension patients.

## 1. Introduction

The morbidity and mortality from coronary heart failure (HF) have recently shown a significant upward trend [1–3]. Coronary HF has become the primary cause of hospitalisation of patients over 65 years worldwide and is currently one of the most serious medical problems [4–7]. The research on the pathogenesis, treatment, and prevention of HF has received increasing attention. Hypertension is a major public health problem worldwide and a significant risk factor for HF. It can be attributed to left ventricular hypertrophy and is an important cause of systolic and diastolic heart failure [8, 9]. Patients with blood pressure (BP) ≥ 160/100 mmHg have a doubled risk of death compared with those with BP < 140/90 mmHg [10].

Autoantibodies are thought to be the result of a dysregulated autoimmune response resulting from the primary disease. These autoantibodies are stable and persistent in the body and not affected by the environment or metabolic pathways. Tissue damage that occurs during the primary disease may result in the release of intracellular proteins that function as autoantigens and thus trigger a humoral response, leading to the production of autoantibodies [11]. The importance of autoimmune mechanisms in the development of cardiac dysfunction has been increasingly recognised. It is believed that abnormal immune activity and multiple immune mechanisms are involved in the pathophysiological process of HF [12]. Cardiac autoantibodies are associated with various pathological states and are a component of the progression of heart disease. Various autoantibodies, such as cardiac myosin, troponin-specific autoantibodies, or muscarinic M2 acetylcholine receptor (M2R), have been detected in the plasma of patients with various cardiac disorders. However, the exact pathophysiological mechanisms of autoimmune-mediated cardiac dysfunction have not been fully elucidated [13]. Studies have found that an autoimmune reaction is one important cause of hypertension [14], and the serum levels of *β*1 adrenergic receptor autoantibodies (*β*1-AA) and angiotensin II type-1 receptor autoantibodies (AT1-AA) [15, 16] in patients with dilated cardiomyopathy (DCM) are higher than those in normal control patients [17].

Wallukat and Wollenberger [18] have found *β*1-AA in the serum of patients with primary cardiomyopathy since 1987, and subsequent studies have shown that *β*1-AA are closely related to various types of cardiomyopathy and HF. *β*1 adrenergic receptor autoantibodies can continuously activate *β*1-adrenergic receptors (*β*1-AR) in cardiomyocytes, resulting in excessive activation of downstream signals, leading to cardiac insufficiency [19]. Removal of serum *β*1-AA by immunoadsorption significantly improved cardiac function [19], highlighting the importance of *β*1-AA in cardiac dysfunction. The literature reports that the detection rate of *β*1-AA in patients with ischemic cardiomyopathy was 10%–55% and in DCM was 26%–95% [20]. Moreover, left ventricular function was significantly reduced in patients with positive levels of *β*1-AA. The study of Chaoyang Hospital in China also found that 45.7% of patients with HF had positive serum *β*1-AA, which was much higher than that of normal control patients (10.4%) [17], and *β*1-AA were positively correlated with maximum ventricular wall thickness and interventricular septal thickness [20].

Angiotensin II type-1 receptor autoantibodies extensively exist in patients with hypertensive diseases [21]. Previous studies have shown that AT1-AA increase BP and synergistically enhance AT1-AA-induced vascular resistance, further aggravating hypertension [22]. Angiotensin II type-1 receptor autoantibodies can increase the concentration of free calcium in cardiomyocytes and enhance the systolic and diastolic functions of the heart, indicating that AT1-AA significantly promote the content of free calcium in cardiomyocytes and cardiac function [23, 24]. Angiotensin II type-1 receptor autoantibodies can be detected in the serum of hypertensive patients with HF, suggesting that they may be involved in the pathological process of myocardial remodelling in HF [25]. Treatment with the angiotensin-converting enzyme inhibitor perindopril significantly reduced AT1-AA and improved left ventricular function and left ventricular remodelling, which further confirms that AT1-AA are one of the important factors in the pathogenesis of hypertension and HF [20].

No previous studies have used autoimmune antibodies as markers for the diagnosis and progression of hypertension. This study is aimed at finding early markers of diastolic dysfunction by observing the relationship between *β*1-AA and AT1-AA and early left ventricular diastolic dysfunction (LVDD) in primary hypertension patients.

## 2. Subjects and Methods

### 2.1. Study Subjects

A total of 120 patients with essential hypertension who were not taking drug treatment and were admitted to the Department of Cardiology at the authors' hospital from June 2022 to December 2022 were enrolled in this study, including 59 males and 61 females aged 18 to 56 (36.73 ± 8.39) years. All patients were aware of their hypertension prior to their admission but did not take antihypertensive medications or other cardiovascular disease-related drugs. Due to the absence of other underlying diseases, such as diabetes, their BP was controlled by diet and lifestyle changes recommended by their physicians.

*Inclusion criteria*: these include patients who met the definition of hypertension in the *2010 Chinese Guidelines for the Prevention and Treatment of Hypertension* and had no previous history of using antihypertensive drugs. Their BP was measured three times on different days, and their systolic blood pressure (SBP) was ≥140 mmHg and/or their diastolic blood pressure (DBP) was ≥90 mmHg. If only their SBP was ≥140 mmHg and their DBP was <90 mmHg, a diagnosis of simple systolic hypertension was made.

*Exclusion criteria*: these include (1) patients with congenital heart disease, valvular heart disease, primary cardiomyopathy, secondary cardiomyopathy other than due to hypertension, chronic obstructive emphysema disease, nonhypertensive-related renal insufficiency, pulmonary hypertension, arrhythmias, and HF with an ejection fraction (EF) of less than 50%; (2) patients with uncontrolled diabetes, hyperlipidemia, or other diseases that have high-risk factors for hypertension; (3) patients with cancer, serious infections, and other serious diseases who were excluded based on history, physical examination, and related auxiliary examination.

**Figure 1:**
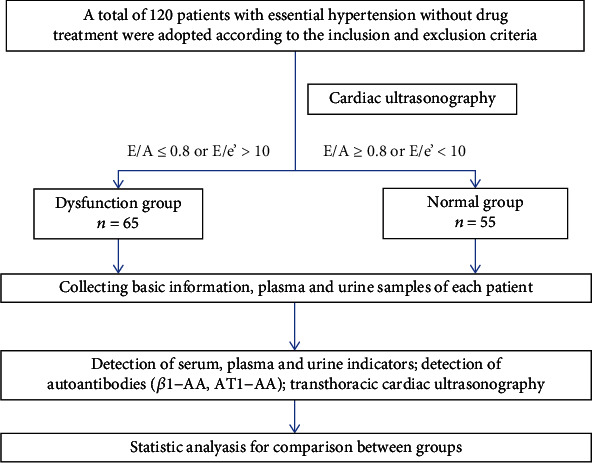
Flow chart of patient enrollment.

The ratio of early diastolic peak blood velocity (*E*) to early diastolic ring motion velocity (*e*′) and the ratio of early diastolic peak blood velocity (*E*) to late diastolic peak blood velocity (*A*) were calculated according to the results of cardiac ultrasonography, with *E*/*A* ≥ 0.8 or *E*/*e*′ < 10 considered normal diastolic function and *E*/*A* < 0.8 or *E*/*e*′ ≥ 10 considered diastolic dysfunction. According to the presence or absence of diastolic cardiac dysfunction, these 120 patients were divided into the dysfunction group (*n* = 65) and the normal group (*n* = 55) (Figure 1).

### 2.2. Data Collection and Indicator Evaluation

After the approval of the Ethics Committee of the authors' hospital and after receiving the informed consent of the patients, the height and weight of the patients were measured, their body mass index (BMI) was calculated, and their resting BP was taken. The patients' BP was measured over 24 h with an ambulatory BP monitor, which took a reading once every 30 min, and their heart rate (HR) was recorded over 24 h with a Holter HR monitor. After admission, a blood test and a random urine sample were taken, and the serum and plasma were centrifuged and stored in a -70°C refrigerator to measure the level of plasma angiotensin II, renin activity, and autoantibodies (*β*1-AA and AT1-AA). The levels of blood glucose, microalbumin (UM), and serum creatinine (Scr) were measured by an automatic biochemical analyser.

#### 2.2.1. Determination of Antibodies (*β*1-AA and AT1-AA)

ELISA kits were used to detect the titers of *β*1-AA (EK714217, AFG Scientific, US) and AT1-AA (A75959-96, Antibodies.com, UK) in the serum of patients [15, 16]. Based on the ratio of the inhalation of positive serum (*P*) to negative serum (*N*), the *P*/*N* ratio was judged, with a *P*/*N* ≥ 2.1 being positive [15, 16] [*P*/*N* = (specimen OD value − blank control OD value)/(negative control OD value − blank control OD value)]. Each measurement had a blank control and a known negative and positive control.

#### 2.2.2. Ultrasonography

Transthoracic cardiac ultrasonography was performed using the Philips ultrasonic diagnostic equipment iE Elite and ultrasonic probe S5-1. Before the examination, the patient was asked to avoid strenuous exercise and eating while being in a stable mood. The patient's information was checked, the light was switched on in the ultrasound room, and the environment was kept quiet. The ultrasound examination bed was located on the left-hand side of the ultrasound equipment, and the height was appropriate for the operating platform. The patient lay on the left side of the examination bed. The patient's left upper limb was extended as far as possible, the chest was leaked, and the intercostal space was expanded as far as possible. The details of the examination were as follows:

1. The left ventricular posterior wall thickness (LVPWd), diastolic ventricular septal thickness (LVSF), and left ventricular end-diastolic diameter (LVEDd) were measured at the left ventricular long-axis section
2. *Left ventricular ejection fraction determination*: Simpson's method was used to measure the EF on the four-chamber incision plane. The images were frozen at the end of the left ventricular diastolic period (the largest ventricular cavity). The endocardial images with relatively clear displays were selected and carefully marked along the endocardial surfaces to measure the left ventricular end-diastolic volume (EDVD4c) of the four-chamber incisal plane. The left ventricular end-systolic section images of the same cardiac cycle were also captured. The left ventricular end-systolic volume (ESVD4c) of the four-chamber incisal plane was recorded, and the EF was obtained by using the Philips iE Elite
3. *Left ventricular short-axis shortening rate (FS)*: the long-axis section of the left ventricle or the short-axis section of the horizontal double ventricle of the papillary muscle was selected, and the sampling line was vertically passed through the right ventricular wall, interventricular septum, and posterior wall of the left ventricle. The left ventricular end-diastolic diameter (Dd), left ventricular end-systolic diameter (Ds), and time of one cardiac cycle (*T*) were measured. Three to five cardiac cycles were measured each time, and the average value was taken. The instrument was imputed, and the FS was calculated using the heart function software program
4. *Early diastolic peak blood velocity (E) to early diastolic mitral annulus velocity (e*′*) and early diastolic peak blood velocity (E) to late diastolic peak blood velocity (A*): the sampling frame was placed at the mitral valve apex on the four-cavity incisal plane of the heart apex. A colour Doppler and pulse Doppler were used to measure the mitral valve velocity. During the measurement, the angle between the direction of the ultrasound beam and the diastolic flow velocity of the mitral valve orifice did not exceed 20° degrees. Both *E*/*A* and *E*/*e*′ were measured

### 2.3. Statistical Analysis

SPSS 24.0 statistical software was used. Measurement data were presented as $\bar{x}\pm s$, and the independent sample *t*-test was used for comparison between the groups. Counting data were expressed as percentages, and the *x*^2^ test was used for comparison between the groups. Multiple regression analysis was used in the multifactor analysis. The variables not associated with hypertension with a *P* < 0.05 using the independent sample *t*-test were included in the multiple regression analysis model. The analysis was adjusted for predetermined variables selected for their potential association with the outcome: age, sex, BMI, smoking, alcohol consumption, EF, FS, and HR. *P* < 0.05 was considered statistically significant.

## 3. Results

### 3.1. General Baseline Characteristics of the Population

In this study, 120 patients with essential hypertension and left ventricular diastolic restriction who were not taking drug treatment were selected, with 55 patients being placed in the normal diastolic function group and 65 patients in the diastolic dysfunction group. There were no statistically significant differences in gender, age, BMI, smoking, and alcohol consumption between the two groups (*P* > 0.05), as Table 1 shows.

### 3.2. Comparison of the Serological Indices, Antibody Levels, and Cardiac Function Parameters between the Two Groups

Table 2 compares the serological indices, antibody levels, and cardiac function parameters between the two groups. Table 2 shows that plasma AngII, plasma renin activity, LVPWd, IVSd, SBP, DBP, *β*1-AA, and AT1-AA in the diastolic dysfunction group were higher than those in the normal group (*P* < 0.05). The two groups had no significant differences in UM, Scr, glucose, LVIDd, EF, FS, and HR (all *P* > 0.05).

### 3.3. Analysis of Influencing Factors on Diastolic Dysfunction

The logistic regression equation takes the reduction of diastolic dysfunction (*E*/*A* ≤ 0.8 or *E*/*e*′ > 10) as the dependent variable and LVPWd, IVSd, SBP, and DBP as the independent variables. The regression analysis results indicated that *β*1-AA and AT1-AA were independent risk factors for cardiac diastolic dysfunction (*P* < 0.05), and the odds ratio values were 1.96 and 2.02, respectively, as Table 3 shows.

### 3.4. Efficacy of *β*1 Adrenergic Receptor Antibodies and Angiotensin II Type-1 Receptor Antibodies and Their Combination in the Diagnosis of Diastolic Dysfunction

Both *β*1-AA and AT1-AA had predictive value for diastolic dysfunction in hypertensive patients (*P* < 0.05), and the area under the curve (AUC) of *β*1-AA for diastolic dysfunction in hypertensive patients was 0.844 (95% confidence interval (CI): 0.772~0.934). The AUC of AT1-AA for predicting diastolic dysfunction in hypertensive patients was 0.841 (95% CI: 0.781~0.869). The combined predicted value of the two indicators was the highest, with an AUC of 0.942 (95% CI: 0.881~0.985). *β*1 adrenergic receptor antibodies combined with AT1-AA predicted that the AUC of diastolic dysfunction in hypertensive patients was higher than *β*1-AA and AT1-AA, and the difference was statistically significant (the *Z* values were 2.13 and 2.42, respectively, *P* < 0.05), as Table 4 shows.

## 4. Discussion

The results of this study were consistent with the previous study, in which autoantibodies to *β*1 and AT1 receptors were detected in the serum of patients with left ventricular diastolic function limitation. The positive results indicate that autoantibodies of various receptors may participate in and affect the pathological process of cardiac structural and functional changes in the occurrence and development of HF. Studies on anti-G-protein-coupled receptor autoantibodies clearly show that the receptor autoantibodies can not only block the binding of receptors and specific ligands but also produce similar agonist effects, act specifically on their respective receptors, and affect the normal regulatory function of their own receptors, thus affecting the structural and functional changes of the heart [26]. Relevant studies have found five main receptors associated with cardiovascular disease: *β*1, *β*2, *α*1 adrenergic, M2R, and AT1 receptors [27, 28]. This study shows that the anti-AT1-antibody and *β*1-receptor immunoglobulin G antibody have a high detection rate in hypertensive patients, especially in patients with substandard BP reduction, suggesting that AT1-receptor and *β*1-receptor antibodies participate in the occurrence and development of hypertension.

Some researchers suggest that detecting *β*1, *β*2, *α*1 adrenergic, M2R, and AT1 receptor autoantibodies in the serum of patients with different heart diseases and HF may have a certain predictive value for ventricular remodelling in different early heart diseases and cardiac function progression [21]. Additionally, it has some reference value for the clinical application of *β*-blockers, converting enzyme inhibitors or receptor antagonists in the treatment of HF [28]. In particular, in patients with positive autoantibodies, the long-term administration of *β*-blockers and converting enzyme inhibitors or angiotensin receptor antagonists to treat HF may have a certain inhibition and reversal effect on the occurrence and development of HF by reducing the role of autoantibodies overstimulating the respective receptors [28]. Basic experiments on the mechanism of action of clinically related drugs and autoantibodies are currently underway [29].

There are some limitations to this study. First, it is a single-centre study, and it is difficult to ensure that the baseline is consistent when the cohort is compared. Second, the indicators of LVDD observed at present are still relatively few, which makes it difficult to fully reflect the actual situation. In addition, the basic research on the relevant mechanism is still limited, and the participating mechanism needs to be further explored and verified. Third, due to the limited time and manpower, the sample size is small, and the representative sample may not be good enough to fully cover the degree of LVDD, which may lead to one-sided results. Finally, future follow-up and interventions with a large sample and multicentre research are planned to further validate the authors' findings.

## 5. Conclusion

The positive rates of autoantibodies of *β*2 and AT1 receptors in the serum of patients with LVDD limitation are high. This suggests that a variety of immune mechanisms may be involved in the pathophysiological process of HF and/or myocardial remodelling, which is worth popularising and applying in a clinical setting.

## Figures and Tables

**Table 1:** Comparison of general data between the two groups.

Group	Dysfunction group (*n* = 65)	Normal group (*n* = 55)	*x* ^2^/*t* value	*P* value
Gender (male/female, *n*)	34/31	25/30	0.560	>0.05
Age (year)	33.22 ± 7.81	34.46 ± 8.18	0.79	>0.05
BMI (kg/m^2^)	23.87 ± 2.1	23.51 ± 2.3	0.84	>0.05
Smoking (*n*, %)	19 (29.23)	15 (27.27)	0.86	>0.05
Alcohol consumption (*n*, %)	17 (26.15)	13 (23.63)	0.75	>0.05

**Table 2:** Comparison of serological indexes, antibody levels, and cardiac function parameters between the two groups.

Characteristic	Dysfunction group (*n* = 65)	Normal group (*n* = 55)	*t*/*x*^2^ value	*P* value
Scr (*μ*mol/L)	78.52 ± 8.98	77.34 ± 8.45	0.72	>0.05
Blood glucose (mmol/L)	4.23 ± 0.44	4.19 ± 0.35	0.32	>0.05
UM (mg/L)	58.23 ± 8.19	12.44 ± 2.55	1.24	>0.05
Plasma AngII (pg/mL)	29.1 ± 1.53	26.75 ± 0.21	2.75	<0.05
Plasma renin activity (ng/mL/hr)	0.63 ± 0.13	0.51 ± 0.13	2.54	<0.05
LVPWd (mm)	10.29 ± 1.23	9.12 ± 1.53	2.47	<0.05
IVSd (mm)	10.56 ± 1.37	9.43 ± 1.44	2.26	<0.05
LVIDd (mm)	47.33 ± 4.35	50.15 ± 3.26	1.88	>0.05
*β*1-AA positive (sample)	33	9	15.501	<0.05
AT1-AA positive (sample)	35	12	12.826	<0.05
EF (%)	65.36 ± 4.75	65.99 ± 5.42	0.37	>0.05
FS (%)	36.35 ± 3.94	35.31 ± 2.91	1.95	>0.05
HR (times/min)	67.33 ± 6.33	67.35 ± 4.57	0.88	>0.05
SBP (mmHg)	152.37 ± 10.24	140.33 ± 5.99	3.56	<0.05
DBP (mmHg)	95.66 ± 6.34	87.33 ± 7.28	4.26	<0.05

Note: UM: microalbumin; LVPWd: left ventricular posterior wall thickness during diastolic period; IVSd: diastolic septal thickness; LVIDd: left ventricular end-diastolic diameter; *β*1-AA: *β*1 adrenergic receptor autoantibody; AT1-AA: autoantibody to angiotensin II1 receptor; EF: ejection fraction; FS: left ventricular short-axis shortening rate; SBP: systolic blood pressure; DBP: diastolic blood pressure.

**Table 3:** Logistic regression analysis of factors influencing diastolic dysfunction in hypertensive patients.

Variable	*β*	S.E.	Wald *x*^2^ value	OR value	*P* value	95% CI
LVPWd	0.48	0.09	6.77	1.55	>0.05	0.669~5.700
IVSd	0.55	0.34	6.53	1.33	>0.05	0.713~2.480
SBP	1.34	0.27	6.44	1.38	>0.05	0.221~4.160
DBP	1.23	0.87	7.61	1.67	>0.05	0.745~3.524
*β*1-AA	0.46	0.33	5.87	1.96	<0.05	1.369~4.345
AT1-AA	0.58	0.61	5.67	2.02	<0.05	1.332~6.720

Note: LVPWd: left ventricular posterior wall thickness during diastolic period; IVSd: diastolic septal thickness; *β*1-AA: *β*1 adrenergic receptor autoantibody; AT1-AA: autoantibody to angiotensin II1 receptor; SBP: systolic blood pressure; DBP: diastolic blood pressure.

**Table 4:** Value of *β*1-AA and AT1-AA and their combination in the prediction of diastolic dysfunction.

Subjects	Accuracy	Sensitivity	Specificity	AUC	95% CI
*β*1-AA	0.860	0.854	0.852	0.844	0.772~0.934
AT1-AA	0.861	0.842	0.813	0.841	0.781~0.869
Combined prediction	0.944	0.931	0.954	0.942	0.881~0.985

Note: *β*1-AA: *β*1 adrenergic receptor autoantibody; AT1-AA: autoantibody to angiotensin II1 receptor.

## Data Availability

The datasets used and analysed during the current study are available from the corresponding author on reasonable request.
